# 2-Fluoro-*N*-(2-fluoro­benzo­yl)-*N*-(2-pyrid­yl)benzamide

**DOI:** 10.1107/S1600536809002189

**Published:** 2009-02-06

**Authors:** John F. Gallagher, Katie Donnelly, Alan J. Lough

**Affiliations:** a School of Chemical Sciences, Dublin City University, Dublin 9, Ireland; b Department of Chemistry, 80 St George Street, University of Toronto, Ontario, Canada M5S 3H6

## Abstract

The title compound, C_19_H_12_F_2_N_2_O_2_, a 2:1 product of the reaction of 2-fluoro­benzoyl chloride and 2-amino­pyridine, crystallizes with a disordered 2-fluoro­benzene ring adopting two conformations [ratio of occupancies = 0.930 (4):0.070 (4)] in one of the two independent mol­ecules (differing slightly in conformation) comprising the asymmetric unit. In the crystal structure, C—H⋯O and C—H⋯π(arene) inter­actions are present.

## Related literature

For background information, see: Donnelly *et al.* (2008 ▶); Gallagher *et al.* (2008 ▶, 2009 ▶); McMahon *et al.* (2008 ▶); Moody *et al.* (1998 ▶). For the parent compound, 2-(dibenzoyl­amino)­pyridine, see: Weng *et al.* (2006 ▶). For related structures, see: Akinboye, Butcher, Brandy *et al.* (2009 ▶); Akinboye, Butcher, Wright *et al.* (2009 ▶); Usman *et al.* (2002*a* ▶,*b* ▶).
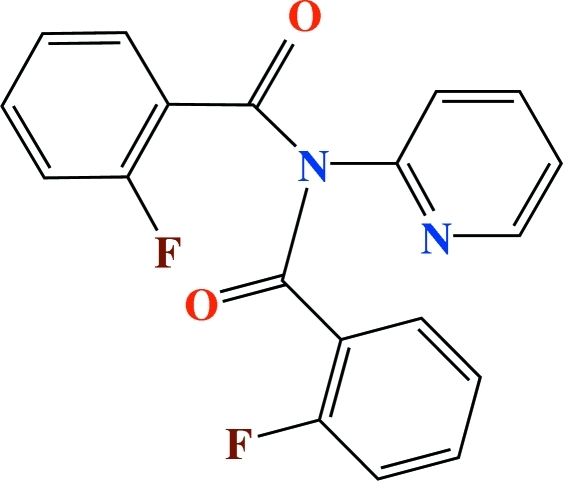


## Experimental

### 

#### Crystal data

C_19_H_12_F_2_N_2_O_2_
*M* *_r_* = 338.31
Monoclinic, 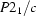

*a* = 8.7421 (3) Å
*b* = 20.4270 (8) Å
*c* = 17.8175 (5) Å
β = 104.145 (2)°
*V* = 3085.29 (18) Å^3^
*Z* = 8
Mo *K*α radiation
μ = 0.11 mm^−1^
*T* = 150 (1) K
0.30 × 0.14 × 0.12 mm

#### Data collection

Nonius KappaCCD diffractometer
Absorption correction: multi-scan (*SORTAV*; Blessing, 1995 ▶) *T*_min_ = 0.850, *T*_max_ = 0.988
7253 measured reflections
7044 independent reflections
3758 reflections with *I* > 2σ(*I*)
*R*_int_ = 0.073

#### Refinement

*R*[*F* ^2^ > 2σ(*F* ^2^)] = 0.054
*wR*(*F* ^2^) = 0.150
*S* = 0.99
7044 reflections
461 parameters
12 restraints
H-atom parameters constrained
Δρ_max_ = 0.29 e Å^−3^
Δρ_min_ = −0.35 e Å^−3^

### 

Data collection: *KappaCCD Server Software* (Nonius, 1997 ▶); cell refinement: *DENZO–SMN* (Otwinowski & Minor, 1997 ▶); data reduction: *DENZO–SMN*; program(s) used to solve structure: *SHELXS97* (Sheldrick, 2008 ▶); program(s) used to refine structure: *SHELXL97* (Sheldrick, 2008 ▶) and *SORTX* (McArdle, 1995 ▶); molecular graphics: *PLATON* (Spek, 2003 ▶); software used to prepare material for publication: *SHELXL97* and *PREP8* (Ferguson, 1998 ▶).

## Supplementary Material

Crystal structure: contains datablocks global, I. DOI: 10.1107/S1600536809002189/tk2358sup1.cif

Structure factors: contains datablocks I. DOI: 10.1107/S1600536809002189/tk2358Isup2.hkl

Additional supplementary materials:  crystallographic information; 3D view; checkCIF report

## Figures and Tables

**Table 1:** Hydrogen-bond geometry (Å, °)

*D*—H⋯*A*	*D*—H	H⋯*A*	*D*⋯*A*	*D*—H⋯*A*
C13*A*—H13*A*⋯O1*B*^i^	0.95	2.41	3.201 (3)	141
C14*A*—H14*A*⋯O2*A*^i^	0.95	2.59	3.493 (3)	158
C16*A*—H16*A*⋯O2*B*	0.95	2.54	3.488 (3)	174
C33*A*—H33*A*⋯O2*B*^ii^	0.95	2.56	3.435 (3)	154
C34*A*—H34*A*⋯O1*A*^ii^	0.95	2.47	3.194 (3)	133
C35*A*—H35*A*⋯*Cg*1^iii^	0.95	2.88	3.591 (3)	133
C16*B*—H16*B*⋯O2*A*	0.95	2.51	3.456 (3)	172
C25*B*—H25*B*⋯O2*B*^iv^	0.95	2.50	3.434 (3)	169

Symmetry codes: (i) 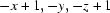; (ii) 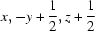; (iii) 
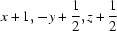
; (iv) 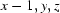. *Cg*1 is the centroid of the C11–C16 benzene ring.

**Fig. 1.**
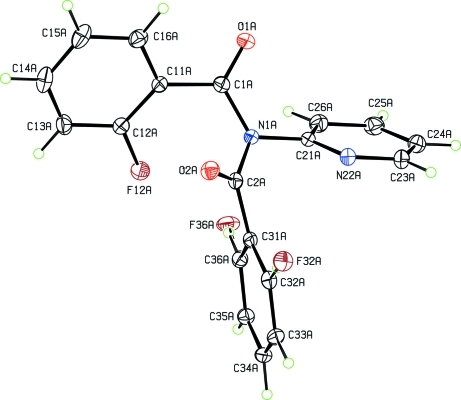
A view of molecule A in (I) with the atomic numbering scheme. The disordered components F32A and F36A are retained. Displacement ellipsoids are drawn at the 30% probability level.

**Fig. 2.**
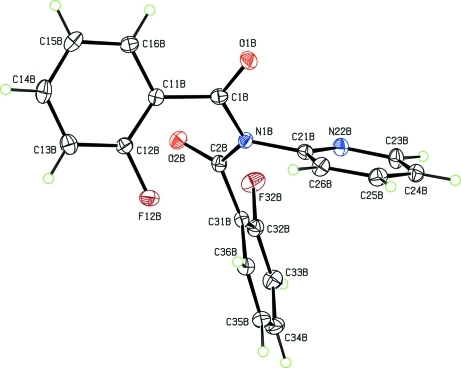
A view of molecule B in (I) with the atomic numbering scheme. Displacement ellipsoids are drawn at the 30% probability level.

**Fig. 3.**
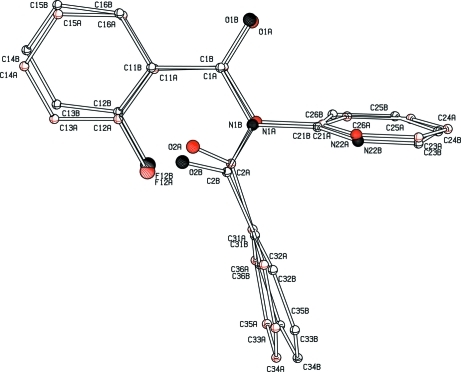
An overlay of molecules A (red) and B (black) in (I) with the disordered F32A/F36A sites and also the F32B atom removed for clarity.

**Fig. 4.**
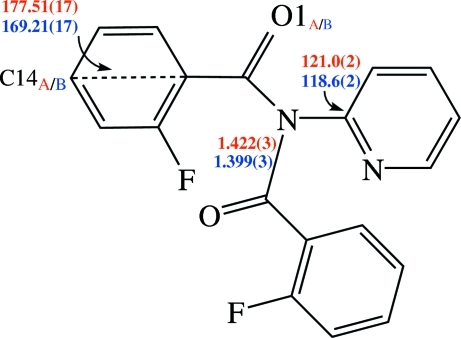
The major differences in bond length and angle between molecules A (red) and B (blue).

## supplementary crystallographic information

### Comment

Our group is completing a structural systematic study of fluoro-*N*'-(pyridyl)benzamide isomers (Donnelly *et al.*, 2008) and we are adding to our research with the analogous difluoro-*N*-(pyridyl)benzamide series (McMahon *et al.*, 2008) (Scheme 1).

In the chemical synthesis of either the mono- or di-fluoro derivatives and when using the *ortho*-aminopyridine, two products can be isolated as either the 1:1 or 2:1 benzoyl:pyridine components and with yields and ratios depending on the reaction conditions. We have reported on the structure of the 1:1 derivative namely 2,3-difluoro-*N*-(2-pyridyl)benzamide (Gallagher *et al.*, 2008) and report herein a 2:1 relative of this type of compound, namely 2-fluoro-*N*-(2-fluorobenzoyl)-*N*-(2-pyridyl)benzamide (I) (Figs 1, 2).

The parent compound 2-(dibenzoylamino)pyridine has been reported previously (Weng *et al.*, 2006) as well as the closely related compounds, *N*,*N*-dibenzoyl-4-chloroaniline and 4-Acetyl-*N*,*N*-dibenzoylphenylamine (Usman *et al.*, 2002*a*,b). A review of the literature suggests that structures of this type are rare despite the large number of substituted benzamides reported. Recently, the crystal structures of two compounds *N*-(3-bromo-1,4-dioxo-1,4-dihydro-2-naphthyl)-2-chloro-*N*-(2-chlorobenzoyl)benzamide & *N*-(3-bromo-1,4-dioxo-1,4-dihydro-2-naphthyl)-4-fluoro-*N*-(4-fluorobenzoyl)benzamide have been reported (Akinboye, Butcher, Brandy *et al.*, 2009; Akinboye, Butcher, Wright *et al.*, 2009) but these differ substantially from (I) in the quinone scaffold or more specifically in the chloro-1,4-naphthoquinone skeleton.

Compound (I) crystallizes with two independent molecules A and B in the asymmetric unit that differ slightly in conformation as depicted in the overlay diagram, Fig. 3. The interesting differences between molecules A and B in (I) can readily be compared with (II) 3-fluoro-*N*-(3-fluorobenzoyl)-*N*-(2-pyridyl)benzamide (Gallagher *et al.*, 2008) and the parent structure 2-(dibenzoylamino)pyridine reported by Weng *et al.* (2006).

In (I), the subtle differences between molecules (A) and (B) are mainly centred about the N1A/N1B tri-substituted C atoms. For example the N1—C2 bond lengths are 1.422 (3) and 1.399 (3) Å, i.e. differ by greater than 0.02 Å, whereas the N1—C1/N1—C21 pair are similar at 1.414 (3)/1.439 (3) Å and 1.419 (3)/1.444 (3) Å in molecules A and B, respectively. The related 3-fluoro structure (II) has bond lengths of 1.420 (3)/1.420 (3)/1.448 (3) Å for the analagous bonds and highlights the short N1B—C2B bond length.

The C1—N1—C2 angles are similar, *i.e.* 121.35 (18)°/121.40 (18)°, but the C1—N1—C21/C2—N1—C21 angle pair are 118.44 (16)°/117.10 (17)° and 116.97 (16)°/120.51 (17)° in A and B, respectively, highlighting both the similarity of the C1—N1—C21/C2—N1—C21 angle pair in A and the 3.5° angle difference in molecule B, as well as the difference in the C2—N1—C21 angle between A and B. The differences in bond lengths and angles must be mainly attributed to crystal packing forces and also the orientational differences of the benzoyl groups at C2A and C2B which can lead to possible and slightly different delocalization along the O2=C2—N1 moiety in A and B. A slight distortion in the N1—C21—C26 angles at 121.0 (2) (A) and 118.6 (2)° (B) is also noted.

The packing distortions also manifest in the C_6_ and C_5_N rings with respect to the central groups to which they are attached. The angles for C1—C11···C14 are 177.51 (17)/169.21 (15)° and the C2—C31···C34 angles are 172.26 (15)/175.25 (16)° in molecules A and B, respectively, highlighting aromatic ring bending differences of > 8° present in the former angle. The corresponding deformation angles in (II) are 176.97 (16) and 174.78 (17)°. In (I), the N1—C21···C24 angles are 177.20 (18) and 179.70 (17)° with little distortion of this central aromatic group with respect to the molecular backbone, Fig. 4.

In the crystal structure, there are no classical hydrogen bonds and interactions comprise weak C—H···O and C—H···π(arene) interactions, Table 1.

### Experimental

Compound (I) was synthesized *via* standard condensation procedures and similar to the related syntheses reported by us (Donnelly *et al.*, 2008; McMahon *et al.*, 2008). Separation of the 1:1 and 2:1 derivatives was undertaken by using flash chromatography using CHCl_3_:ethyl acetate. Typical organic workup and washing gave the product (I) in modest yield of 30–40% as a 2:1 component of the mixture. Crystals suitable for X-ray diffraction were grown from CHCl_3_ as colourless blocks over a period of 1–2 weeks and gave a melting point of 367–371 K. The compounds gave clean ^1^H and ^13^C NMR spectra in CDCl_3_ solution. IR (ν_C=O_ cm^-1^): 1715, 1688(*s, br*), (CHCl_3_); 1710, 1698(*s*) (KBr).

### Refinement

In the final stages of refinement it was observed that there was electron density consistent with a partial occupancy F atom in a position expected for a minor orientation (site) of the F32A F atom position as F36A. This new site only necessitates rotation by 180° about the C2A—C31A axis in a group that is not engaged in strong hydrogen bonding and is relatively free to rotate.

The minor F36A site was treated initially with isotropic displacement values and in the final refinement cycles was restrained by DELU/ISOR restraints of 0.1 (for F32A, F36A). The final refinement cycles gave site occupancy values of 0.930 (4):0.070 (4). As the major and minor sites for the C_6_ ring essentially coincide it was decided to retain the major orientation with 100% occupancy for use with the restraints.

The H atoms attached to C atoms were treated as riding with C—H = 0.95 Å, and with U_iso_(H) = 1.2U_eq_(C).

### Crystal data

**Table:**

C_19_H_12_F_2_N_2_O_2_	*F*(000) = 1392
*M**_r_* = 338.31	*D*_x_ = 1.457 Mg m^−^^3^
Monoclinic, *P*2_1_/*c*	Melting point: 367 K
Hall symbol: -P 2ybc	Mo *K*α radiation, λ = 0.71073 Å
*a* = 8.7421 (3) Å	Cell parameters from 8844 reflections
*b* = 20.4270 (8) Å	θ = 2.6–27.5°
*c* = 17.8175 (5) Å	µ = 0.11 mm^−^^1^
β = 104.145 (2)°	*T* = 150 K
*V* = 3085.29 (18) Å^3^	Block, colourless
*Z* = 8	0.30 × 0.14 × 0.12 mm

### Data collection

**Table:**

Nonius KappaCCD diffractometer	7044 independent reflections
Radiation source: fine-focus sealed X-ray tube	3758 reflections with *I* > 2σ(*I*)
graphite	*R*_int_ = 0.073
φ, and ω scans with κ offsets	θ_max_ = 27.5°, θ_min_ = 2.6°
Absorption correction: multi-scan (*SORTAV*; Blessing, 1995)	*h* = −11→10
*T*_min_ = 0.850, *T*_max_ = 0.988	*k* = −26→26
7253 measured reflections	*l* = −19→23

### Refinement

**Table:**

Refinement on *F*^2^	Primary atom site location: structure-invariant direct methods
Least-squares matrix: full	Secondary atom site location: difference Fourier map
*R*[*F*^2^ > 2σ(*F*^2^)] = 0.054	Hydrogen site location: inferred from neighbouring sites
*wR*(*F*^2^) = 0.150	H-atom parameters constrained
*S* = 0.99	*w* = 1/[σ^2^(*F*_o_^2^) + (0.0741*P*)^2^] where *P* = (*F*_o_^2^ + 2*F*_c_^2^)/3
7044 reflections	(Δ/σ)_max_ < 0.001
461 parameters	Δρ_max_ = 0.29 e Å^−^^3^
12 restraints	Δρ_min_ = −0.35 e Å^−^^3^

### Fractional atomic coordinates and isotropic or equivalent isotropic displacement parameters (Å^2^)

**Table:**

	*x*	*y*	*z*	*U*_iso_*/*U*_eq_	Occ. (<1)
F12A	0.92767 (18)	0.06141 (7)	0.57392 (7)	0.0448 (4)	
O1A	0.9098 (2)	0.11920 (8)	0.36102 (9)	0.0388 (5)	
C1A	0.8666 (3)	0.11698 (11)	0.42060 (12)	0.0275 (5)	
N1A	0.8933 (2)	0.17057 (9)	0.47236 (10)	0.0253 (4)	
C11A	0.7887 (3)	0.05768 (11)	0.44252 (12)	0.0258 (5)	
C12A	0.8199 (3)	0.03140 (11)	0.51633 (13)	0.0297 (6)	
C13A	0.7479 (3)	−0.02385 (12)	0.53475 (14)	0.0359 (6)	
C14A	0.6365 (3)	−0.05401 (13)	0.47674 (17)	0.0418 (7)	
C15A	0.6029 (3)	−0.03026 (13)	0.40146 (16)	0.0420 (7)	
C16A	0.6804 (3)	0.02463 (12)	0.38468 (14)	0.0342 (6)	
C21A	0.9919 (3)	0.22331 (11)	0.45829 (11)	0.0236 (5)	
N22A	0.9235 (2)	0.28159 (9)	0.45101 (10)	0.0287 (5)	
C23A	1.0123 (3)	0.33207 (12)	0.43746 (13)	0.0342 (6)	
C24A	1.1639 (3)	0.32577 (13)	0.42965 (13)	0.0349 (6)	
C25A	1.2317 (3)	0.26415 (13)	0.43804 (13)	0.0352 (6)	
C26A	1.1448 (3)	0.21177 (12)	0.45259 (13)	0.0321 (6)	
O2A	0.65467 (19)	0.16567 (8)	0.50477 (8)	0.0318 (4)	
C2A	0.7900 (3)	0.18421 (11)	0.52090 (12)	0.0245 (5)	
C31A	0.8623 (3)	0.21946 (11)	0.59384 (12)	0.0232 (5)	
C32A	0.7764 (3)	0.26714 (11)	0.62237 (12)	0.0265 (5)	
C33A	0.8292 (3)	0.29479 (11)	0.69417 (13)	0.0298 (6)	
C34A	0.9731 (3)	0.27489 (12)	0.74019 (13)	0.0296 (6)	
C35A	1.0635 (3)	0.22887 (11)	0.71327 (12)	0.0270 (5)	
C36A	1.0083 (3)	0.20202 (11)	0.64032 (13)	0.0269 (5)	
F32A	0.63827 (17)	0.28808 (7)	0.57553 (8)	0.0344 (5)	0.930 (4)
F36A	1.116 (2)	0.1620 (10)	0.6169 (10)	0.039 (7)	0.070 (4)
F12B	0.31068 (16)	0.18071 (7)	0.12221 (7)	0.0369 (4)	
O1B	0.3159 (2)	0.12056 (8)	0.33481 (8)	0.0338 (4)	
C1B	0.3604 (3)	0.12421 (11)	0.27555 (12)	0.0255 (5)	
N1B	0.3322 (2)	0.07118 (9)	0.22248 (10)	0.0226 (4)	
C11B	0.4330 (3)	0.18597 (11)	0.25606 (12)	0.0228 (5)	
C12B	0.3982 (3)	0.21466 (11)	0.18366 (12)	0.0261 (5)	
C13B	0.4425 (3)	0.27768 (12)	0.17050 (14)	0.0331 (6)	
C14B	0.5283 (3)	0.31302 (12)	0.23274 (15)	0.0369 (6)	
C15B	0.5697 (3)	0.28541 (12)	0.30609 (14)	0.0343 (6)	
C16B	0.5208 (3)	0.22290 (12)	0.31764 (12)	0.0276 (6)	
O2B	0.57182 (18)	0.07792 (8)	0.19299 (8)	0.0287 (4)	
C2B	0.4384 (3)	0.05560 (11)	0.17782 (12)	0.0226 (5)	
C21B	0.2073 (3)	0.02639 (11)	0.22749 (12)	0.0240 (5)	
N22B	0.2476 (2)	−0.03583 (9)	0.23967 (10)	0.0266 (5)	
C23B	0.1297 (3)	−0.07729 (12)	0.24352 (13)	0.0303 (6)	
C24B	−0.0238 (3)	−0.05737 (12)	0.23712 (13)	0.0297 (6)	
C25B	−0.0610 (3)	0.00756 (12)	0.22546 (13)	0.0313 (6)	
C26B	0.0564 (3)	0.05108 (12)	0.21966 (13)	0.0282 (6)	
F32B	0.59097 (16)	−0.05992 (7)	0.15023 (8)	0.0399 (4)	
C31B	0.3775 (3)	0.01289 (11)	0.10932 (12)	0.0231 (5)	
C32B	0.4601 (3)	−0.04192 (11)	0.09556 (13)	0.0268 (5)	
C33B	0.4133 (3)	−0.07992 (12)	0.03030 (14)	0.0329 (6)	
C34B	0.2801 (3)	−0.06149 (12)	−0.02500 (13)	0.0322 (6)	
C35B	0.1943 (3)	−0.00704 (12)	−0.01392 (13)	0.0301 (6)	
C36B	0.2418 (3)	0.02922 (12)	0.05330 (12)	0.0272 (5)	
H13A	0.7738	−0.0409	0.5859	0.043*	
H14A	0.5822	−0.0915	0.4884	0.050*	
H15A	0.5271	−0.0517	0.3617	0.050*	
H16A	0.6595	0.0401	0.3329	0.041*	
H23A	0.9672	0.3746	0.4330	0.041*	
H24A	1.2208	0.3627	0.4188	0.042*	
H25A	1.3370	0.2582	0.4338	0.042*	
H26A	1.1882	0.1689	0.4586	0.039*	
H32A	0.6778	0.2809	0.5908	0.032*	0.070 (4)
H33A	0.7682	0.3270	0.7120	0.036*	
H34A	1.0101	0.2929	0.7905	0.036*	
H35A	1.1628	0.2158	0.7448	0.032*	
H36A	1.0716	0.1711	0.6218	0.032*	0.930 (4)
H13B	0.4148	0.2962	0.1201	0.040*	
H14B	0.5593	0.3566	0.2253	0.044*	
H15B	0.6316	0.3096	0.3483	0.041*	
H16B	0.5472	0.2047	0.3682	0.033*	
H23B	0.1536	−0.1226	0.2510	0.036*	
H24B	−0.1027	−0.0883	0.2408	0.036*	
H25B	−0.1657	0.0224	0.2214	0.038*	
H26B	0.0347	0.0964	0.2106	0.034*	
H33B	0.4711	−0.1179	0.0234	0.039*	
H34B	0.2472	−0.0865	−0.0711	0.039*	
H35B	0.1030	0.0054	−0.0523	0.036*	
H36B	0.1809	0.0659	0.0614	0.033*	

### Atomic displacement parameters (Å^2^)

**Table:**

	*U*^11^	*U*^22^	*U*^33^	*U*^12^	*U*^13^	*U*^23^
F12A	0.0657 (11)	0.0363 (9)	0.0259 (7)	−0.0103 (8)	−0.0013 (7)	0.0017 (6)
O1A	0.0593 (13)	0.0352 (11)	0.0259 (9)	−0.0102 (9)	0.0179 (8)	−0.0037 (8)
C1A	0.0307 (14)	0.0275 (14)	0.0235 (12)	−0.0019 (11)	0.0050 (10)	0.0008 (10)
N1A	0.0333 (12)	0.0219 (11)	0.0227 (10)	−0.0053 (9)	0.0106 (8)	−0.0023 (8)
C11A	0.0302 (14)	0.0217 (13)	0.0266 (12)	−0.0002 (11)	0.0089 (10)	−0.0014 (10)
C12A	0.0373 (15)	0.0245 (14)	0.0269 (12)	−0.0012 (12)	0.0074 (11)	−0.0011 (10)
C13A	0.0472 (17)	0.0249 (14)	0.0397 (14)	0.0030 (13)	0.0186 (13)	0.0065 (11)
C14A	0.0358 (16)	0.0250 (15)	0.0669 (19)	−0.0029 (12)	0.0169 (14)	0.0046 (13)
C15A	0.0334 (16)	0.0279 (16)	0.0592 (18)	−0.0049 (12)	0.0006 (13)	−0.0053 (13)
C16A	0.0368 (15)	0.0296 (15)	0.0324 (13)	0.0016 (12)	0.0009 (11)	−0.0026 (11)
C21A	0.0315 (14)	0.0205 (13)	0.0194 (11)	−0.0035 (11)	0.0071 (10)	−0.0006 (9)
N22A	0.0347 (12)	0.0244 (12)	0.0278 (10)	−0.0027 (9)	0.0092 (9)	0.0010 (8)
C23A	0.0446 (17)	0.0252 (14)	0.0323 (13)	−0.0037 (12)	0.0084 (11)	0.0034 (11)
C24A	0.0375 (16)	0.0368 (17)	0.0309 (13)	−0.0140 (13)	0.0095 (11)	−0.0027 (11)
C25A	0.0318 (15)	0.0418 (17)	0.0349 (14)	−0.0049 (13)	0.0137 (11)	−0.0055 (12)
C26A	0.0338 (15)	0.0306 (15)	0.0330 (13)	0.0002 (12)	0.0103 (11)	−0.0019 (11)
O2A	0.0271 (10)	0.0336 (10)	0.0347 (9)	−0.0057 (8)	0.0073 (7)	−0.0030 (7)
C2A	0.0285 (14)	0.0199 (13)	0.0254 (12)	0.0004 (11)	0.0074 (10)	0.0025 (9)
C31A	0.0254 (13)	0.0214 (13)	0.0252 (11)	−0.0034 (10)	0.0107 (10)	0.0006 (9)
C32A	0.0248 (13)	0.0260 (14)	0.0289 (12)	−0.0008 (11)	0.0070 (10)	0.0041 (10)
C33A	0.0383 (16)	0.0238 (14)	0.0308 (13)	0.0015 (11)	0.0148 (11)	−0.0040 (10)
C34A	0.0370 (15)	0.0306 (15)	0.0232 (12)	−0.0077 (12)	0.0109 (11)	−0.0041 (10)
C35A	0.0279 (14)	0.0279 (14)	0.0249 (12)	−0.0020 (11)	0.0056 (10)	−0.0006 (10)
C36A	0.0297 (14)	0.0236 (13)	0.0305 (12)	−0.0014 (11)	0.0134 (11)	−0.0013 (10)
F32A	0.0323 (10)	0.0337 (10)	0.0364 (9)	0.0058 (7)	0.0067 (7)	0.0020 (7)
F36A	0.049 (10)	0.041 (10)	0.028 (9)	0.007 (7)	0.009 (7)	−0.010 (7)
F12B	0.0475 (9)	0.0340 (9)	0.0248 (7)	−0.0040 (7)	0.0002 (6)	0.0016 (6)
O1B	0.0453 (11)	0.0324 (10)	0.0273 (9)	−0.0073 (8)	0.0159 (8)	−0.0026 (7)
C1B	0.0245 (13)	0.0264 (14)	0.0246 (12)	0.0022 (11)	0.0040 (10)	−0.0003 (10)
N1B	0.0238 (11)	0.0220 (11)	0.0236 (10)	−0.0032 (8)	0.0087 (8)	−0.0031 (8)
C11B	0.0220 (12)	0.0199 (13)	0.0272 (12)	0.0028 (10)	0.0073 (10)	−0.0008 (10)
C12B	0.0262 (14)	0.0254 (14)	0.0259 (12)	−0.0004 (10)	0.0048 (10)	−0.0012 (10)
C13B	0.0346 (15)	0.0286 (15)	0.0373 (14)	0.0027 (12)	0.0112 (12)	0.0057 (11)
C14B	0.0344 (16)	0.0231 (15)	0.0543 (17)	−0.0028 (12)	0.0130 (13)	0.0028 (12)
C15B	0.0288 (15)	0.0289 (15)	0.0428 (15)	−0.0039 (11)	0.0043 (11)	−0.0089 (11)
C16B	0.0258 (13)	0.0291 (14)	0.0267 (12)	0.0024 (11)	0.0041 (10)	−0.0017 (10)
O2B	0.0230 (10)	0.0303 (10)	0.0324 (9)	−0.0024 (8)	0.0061 (7)	−0.0030 (7)
C2B	0.0226 (13)	0.0181 (12)	0.0266 (12)	0.0027 (10)	0.0051 (10)	0.0035 (9)
C21B	0.0282 (14)	0.0243 (14)	0.0205 (11)	−0.0017 (11)	0.0081 (9)	−0.0014 (9)
N22B	0.0271 (11)	0.0230 (12)	0.0300 (10)	−0.0004 (9)	0.0076 (8)	0.0016 (8)
C23B	0.0314 (15)	0.0214 (13)	0.0385 (14)	−0.0021 (11)	0.0093 (11)	0.0051 (11)
C24B	0.0265 (14)	0.0324 (15)	0.0302 (13)	−0.0038 (11)	0.0066 (10)	0.0026 (11)
C25B	0.0247 (14)	0.0377 (16)	0.0320 (13)	0.0000 (12)	0.0077 (10)	−0.0031 (11)
C26B	0.0269 (14)	0.0223 (13)	0.0350 (13)	0.0041 (11)	0.0069 (10)	−0.0016 (10)
F32B	0.0325 (8)	0.0372 (9)	0.0440 (8)	0.0094 (7)	−0.0021 (7)	−0.0055 (7)
C31B	0.0249 (13)	0.0213 (13)	0.0247 (11)	−0.0005 (10)	0.0092 (10)	0.0012 (9)
C32B	0.0214 (13)	0.0289 (14)	0.0294 (12)	−0.0007 (11)	0.0050 (10)	0.0001 (10)
C33B	0.0351 (16)	0.0282 (14)	0.0369 (14)	0.0022 (12)	0.0117 (12)	−0.0081 (11)
C34B	0.0336 (15)	0.0353 (16)	0.0276 (13)	−0.0044 (12)	0.0076 (11)	−0.0082 (11)
C35B	0.0271 (14)	0.0335 (15)	0.0273 (12)	−0.0007 (12)	0.0020 (10)	0.0016 (11)
C36B	0.0239 (13)	0.0298 (14)	0.0282 (12)	0.0026 (11)	0.0070 (10)	−0.0006 (10)

### Geometric parameters (Å, °)

**Table:**

F12A—C12A	1.358 (3)	F12B—C12B	1.362 (2)
O1A—C1A	1.211 (2)	O1B—C1B	1.214 (2)
C1A—N1A	1.414 (3)	C1B—N1B	1.419 (3)
C1A—C11A	1.488 (3)	C1B—C11B	1.491 (3)
N1A—C2A	1.422 (3)	N1B—C2B	1.399 (3)
N1A—C21A	1.439 (3)	N1B—C21B	1.444 (3)
C11A—C12A	1.385 (3)	C11B—C12B	1.381 (3)
C11A—C16A	1.392 (3)	C11B—C16B	1.397 (3)
C12A—C13A	1.371 (3)	C12B—C13B	1.381 (3)
C13A—C14A	1.380 (4)	C13B—C14B	1.381 (3)
C13A—H13A	0.9500	C13B—H13B	0.9500
C14A—C15A	1.389 (4)	C14B—C15B	1.388 (3)
C14A—H14A	0.9500	C14B—H14B	0.9500
C15A—C16A	1.380 (3)	C15B—C16B	1.378 (3)
C15A—H15A	0.9500	C15B—H15B	0.9500
C16A—H16A	0.9500	C16B—H16B	0.9500
O2A—C2A	1.208 (3)	O2B—C2B	1.220 (3)
C2A—C31A	1.485 (3)	C2B—C31B	1.489 (3)
C21A—N22A	1.324 (3)	C21B—N22B	1.323 (3)
C21A—C26A	1.385 (3)	C21B—C26B	1.387 (3)
N22A—C23A	1.348 (3)	N22B—C23B	1.349 (3)
C23A—C24A	1.372 (4)	C23B—C24B	1.380 (3)
C23A—H23A	0.9500	C23B—H23B	0.9500
C24A—C25A	1.384 (3)	C24B—C25B	1.369 (3)
C24A—H24A	0.9500	C24B—H24B	0.9500
C25A—C26A	1.373 (3)	C25B—C26B	1.381 (3)
C25A—H25A	0.9500	C25B—H25B	0.9500
C26A—H26A	0.9500	C26B—H26B	0.9500
C31A—C36A	1.388 (3)	F32B—C32B	1.360 (3)
C31A—C32A	1.399 (3)	C31B—C32B	1.386 (3)
C32A—C33A	1.371 (3)	C31B—C36B	1.391 (3)
C32A—H32A	0.9500	C32B—C33B	1.374 (3)
C33A—C34A	1.384 (3)	C33B—C34B	1.381 (3)
C33A—H33A	0.9500	C33B—H33B	0.9500
C34A—C35A	1.387 (3)	C34B—C35B	1.383 (3)
C34A—H34A	0.9500	C34B—H34B	0.9500
C35A—C36A	1.384 (3)	C35B—C36B	1.383 (3)
C35A—H35A	0.9500	C35B—H35B	0.9500
C36A—H36A	0.9500	C36B—H36B	0.9500
			
O1A—C1A—N1A	120.4 (2)	O1B—C1B—N1B	119.5 (2)
O1A—C1A—C11A	121.4 (2)	O1B—C1B—C11B	119.99 (19)
N1A—C1A—C11A	118.18 (18)	N1B—C1B—C11B	120.36 (18)
C1A—N1A—C2A	121.35 (18)	C2B—N1B—C1B	121.40 (18)
C1A—N1A—C21A	118.44 (16)	C2B—N1B—C21B	120.51 (17)
C2A—N1A—C21A	117.10 (17)	C1B—N1B—C21B	116.97 (16)
C12A—C11A—C16A	117.1 (2)	C12B—C11B—C16B	117.3 (2)
C12A—C11A—C1A	124.6 (2)	C12B—C11B—C1B	124.6 (2)
C16A—C11A—C1A	118.2 (2)	C16B—C11B—C1B	117.23 (19)
F12A—C12A—C13A	117.7 (2)	F12B—C12B—C13B	117.6 (2)
F12A—C12A—C11A	118.8 (2)	F12B—C12B—C11B	119.1 (2)
C13A—C12A—C11A	123.4 (2)	C13B—C12B—C11B	123.2 (2)
C12A—C13A—C14A	118.0 (2)	C14B—C13B—C12B	118.1 (2)
C12A—C13A—H13A	121.0	C14B—C13B—H13B	121.0
C14A—C13A—H13A	121.0	C12B—C13B—H13B	121.0
C13A—C14A—C15A	120.8 (2)	C13B—C14B—C15B	120.6 (2)
C13A—C14A—H14A	119.6	C13B—C14B—H14B	119.7
C15A—C14A—H14A	119.6	C15B—C14B—H14B	119.7
C16A—C15A—C14A	119.6 (2)	C16B—C15B—C14B	119.9 (2)
C16A—C15A—H15A	120.2	C16B—C15B—H15B	120.1
C14A—C15A—H15A	120.2	C14B—C15B—H15B	120.1
C15A—C16A—C11A	121.0 (2)	C15B—C16B—C11B	120.9 (2)
C15A—C16A—H16A	119.5	C15B—C16B—H16B	119.5
C11A—C16A—H16A	119.5	C11B—C16B—H16B	119.5
N22A—C21A—C26A	124.5 (2)	O2B—C2B—N1B	121.36 (19)
N22A—C21A—N1A	114.50 (19)	O2B—C2B—C31B	122.12 (19)
C26A—C21A—N1A	121.0 (2)	N1B—C2B—C31B	116.47 (19)
C21A—N22A—C23A	116.1 (2)	N22B—C21B—C26B	125.1 (2)
N22A—C23A—C24A	124.0 (2)	N22B—C21B—N1B	116.36 (19)
N22A—C23A—H23A	118.0	C26B—C21B—N1B	118.6 (2)
C24A—C23A—H23A	118.0	C21B—N22B—C23B	115.8 (2)
C23A—C24A—C25A	118.3 (2)	N22B—C23B—C24B	123.4 (2)
C23A—C24A—H24A	120.9	N22B—C23B—H23B	118.3
C25A—C24A—H24A	120.9	C24B—C23B—H23B	118.3
C26A—C25A—C24A	119.1 (2)	C25B—C24B—C23B	119.2 (2)
C26A—C25A—H25A	120.4	C25B—C24B—H24B	120.4
C24A—C25A—H25A	120.4	C23B—C24B—H24B	120.4
C25A—C26A—C21A	118.0 (2)	C24B—C25B—C26B	118.8 (2)
C25A—C26A—H26A	121.0	C24B—C25B—H25B	120.6
C21A—C26A—H26A	121.0	C26B—C25B—H25B	120.6
O2A—C2A—N1A	121.6 (2)	C25B—C26B—C21B	117.7 (2)
O2A—C2A—C31A	122.89 (19)	C25B—C26B—H26B	121.2
N1A—C2A—C31A	115.42 (19)	C21B—C26B—H26B	121.2
C36A—C31A—C32A	117.3 (2)	C32B—C31B—C36B	117.2 (2)
C36A—C31A—C2A	122.0 (2)	C32B—C31B—C2B	121.4 (2)
C32A—C31A—C2A	120.3 (2)	C36B—C31B—C2B	121.3 (2)
C33A—C32A—C31A	122.5 (2)	F32B—C32B—C33B	118.5 (2)
C33A—C32A—H32A	118.7	F32B—C32B—C31B	118.4 (2)
C31A—C32A—H32A	118.7	C33B—C32B—C31B	123.1 (2)
C32A—C33A—C34A	118.8 (2)	C32B—C33B—C34B	118.3 (2)
C32A—C33A—H33A	120.6	C32B—C33B—H33B	120.8
C34A—C33A—H33A	120.6	C34B—C33B—H33B	120.8
C33A—C34A—C35A	120.4 (2)	C33B—C34B—C35B	120.6 (2)
C33A—C34A—H34A	119.8	C33B—C34B—H34B	119.7
C35A—C34A—H34A	119.8	C35B—C34B—H34B	119.7
C36A—C35A—C34A	119.8 (2)	C34B—C35B—C36B	119.7 (2)
C36A—C35A—H35A	120.1	C34B—C35B—H35B	120.1
C34A—C35A—H35A	120.1	C36B—C35B—H35B	120.1
C35A—C36A—C31A	121.1 (2)	C35B—C36B—C31B	121.1 (2)
C35A—C36A—H36A	119.4	C35B—C36B—H36B	119.5
C31A—C36A—H36A	119.4	C31B—C36B—H36B	119.5
			
O1A—C1A—N1A—C2A	150.7 (2)	O1B—C1B—N1B—C21B	21.0 (3)
C11A—C1A—N1A—C2A	−31.0 (3)	C11B—C1B—N1B—C21B	−154.2 (2)
O1A—C1A—N1A—C21A	−8.8 (3)	O1B—C1B—C11B—C12B	−135.7 (2)
C11A—C1A—N1A—C21A	169.5 (2)	N1B—C1B—C11B—C12B	39.5 (3)
O1A—C1A—C11A—C12A	137.7 (2)	O1B—C1B—C11B—C16B	32.8 (3)
N1A—C1A—C11A—C12A	−40.6 (3)	N1B—C1B—C11B—C16B	−152.0 (2)
O1A—C1A—C11A—C16A	−40.0 (3)	C16B—C11B—C12B—F12B	−178.99 (18)
N1A—C1A—C11A—C16A	141.7 (2)	C1B—C11B—C12B—F12B	−10.5 (3)
C16A—C11A—C12A—F12A	178.5 (2)	C16B—C11B—C12B—C13B	−1.7 (3)
C1A—C11A—C12A—F12A	0.8 (3)	C1B—C11B—C12B—C13B	166.9 (2)
C16A—C11A—C12A—C13A	−1.4 (4)	F12B—C12B—C13B—C14B	178.6 (2)
C1A—C11A—C12A—C13A	−179.1 (2)	C11B—C12B—C13B—C14B	1.2 (4)
F12A—C12A—C13A—C14A	179.0 (2)	C12B—C13B—C14B—C15B	0.7 (4)
C11A—C12A—C13A—C14A	−1.1 (4)	C13B—C14B—C15B—C16B	−2.0 (4)
C12A—C13A—C14A—C15A	2.2 (4)	C14B—C15B—C16B—C11B	1.6 (4)
C13A—C14A—C15A—C16A	−0.8 (4)	C12B—C11B—C16B—C15B	0.2 (3)
C14A—C15A—C16A—C11A	−1.8 (4)	C1B—C11B—C16B—C15B	−169.2 (2)
C12A—C11A—C16A—C15A	2.8 (4)	C1B—N1B—C2B—O2B	14.6 (3)
C1A—C11A—C16A—C15A	−179.3 (2)	C21B—N1B—C2B—O2B	−153.0 (2)
C1A—N1A—C21A—N22A	123.0 (2)	C1B—N1B—C2B—C31B	−162.91 (19)
C2A—N1A—C21A—N22A	−37.3 (3)	C21B—N1B—C2B—C31B	29.5 (3)
C1A—N1A—C21A—C26A	−56.5 (3)	C2B—N1B—C21B—N22B	45.8 (3)
C2A—N1A—C21A—C26A	143.2 (2)	C1B—N1B—C21B—N22B	−122.3 (2)
C26A—C21A—N22A—C23A	−0.3 (3)	C2B—N1B—C21B—C26B	−134.4 (2)
N1A—C21A—N22A—C23A	−179.74 (18)	C1B—N1B—C21B—C26B	57.5 (3)
C21A—N22A—C23A—C24A	1.3 (3)	C26B—C21B—N22B—C23B	0.9 (3)
N22A—C23A—C24A—C25A	−1.7 (4)	N1B—C21B—N22B—C23B	−179.40 (18)
C23A—C24A—C25A—C26A	1.0 (3)	C21B—N22B—C23B—C24B	−1.4 (3)
C24A—C25A—C26A—C21A	−0.1 (3)	N22B—C23B—C24B—C25B	0.7 (3)
N22A—C21A—C26A—C25A	−0.3 (3)	C23B—C24B—C25B—C26B	0.7 (3)
N1A—C21A—C26A—C25A	179.12 (19)	C24B—C25B—C26B—C21B	−1.2 (3)
C1A—N1A—C2A—O2A	−23.5 (3)	N22B—C21B—C26B—C25B	0.4 (3)
C21A—N1A—C2A—O2A	136.3 (2)	N1B—C21B—C26B—C25B	−179.31 (18)
C1A—N1A—C2A—C31A	153.5 (2)	O2B—C2B—C31B—C32B	51.8 (3)
C21A—N1A—C2A—C31A	−46.8 (3)	N1B—C2B—C31B—C32B	−130.8 (2)
O2A—C2A—C31A—C36A	132.2 (2)	O2B—C2B—C31B—C36B	−123.1 (2)
N1A—C2A—C31A—C36A	−44.8 (3)	N1B—C2B—C31B—C36B	54.4 (3)
O2A—C2A—C31A—C32A	−40.7 (3)	C36B—C31B—C32B—F32B	−178.40 (19)
N1A—C2A—C31A—C32A	142.4 (2)	C2B—C31B—C32B—F32B	6.5 (3)
C36A—C31A—C32A—C33A	−2.1 (3)	C36B—C31B—C32B—C33B	−0.3 (3)
C2A—C31A—C32A—C33A	171.1 (2)	C2B—C31B—C32B—C33B	−175.4 (2)
C31A—C32A—C33A—C34A	0.1 (3)	F32B—C32B—C33B—C34B	179.9 (2)
C32A—C33A—C34A—C35A	1.4 (3)	C31B—C32B—C33B—C34B	1.8 (4)
C33A—C34A—C35A—C36A	−0.9 (3)	C32B—C33B—C34B—C35B	−1.5 (4)
C34A—C35A—C36A—C31A	−1.2 (3)	C33B—C34B—C35B—C36B	−0.2 (4)
C32A—C31A—C36A—C35A	2.6 (3)	C34B—C35B—C36B—C31B	1.8 (3)
C2A—C31A—C36A—C35A	−170.4 (2)	C32B—C31B—C36B—C35B	−1.5 (3)
O1B—C1B—N1B—C2B	−147.0 (2)	C2B—C31B—C36B—C35B	173.6 (2)
C11B—C1B—N1B—C2B	37.8 (3)		

### Hydrogen-bond geometry (Å, °)

**Table:**

*D*—H···*A*	*D*—H	H···*A*	*D*···*A*	*D*—H···*A*
C13A—H13A···O1B^i^	0.95	2.41	3.201 (3)	141
C14A—H14A···O2A^i^	0.95	2.59	3.493 (3)	158
C16A—H16A···O2B	0.95	2.54	3.488 (3)	174
C33A—H33A···O2B^ii^	0.95	2.56	3.435 (3)	154
C34A—H34A···O1A^ii^	0.95	2.47	3.194 (3)	133
C35A—H35A···Cg1^iii^	0.95	2.88	3.591 (3)	133
C16B—H16B···O2A	0.95	2.51	3.456 (3)	172
C25B—H25B···O2B^iv^	0.95	2.50	3.434 (3)	169

Symmetry codes: (i) −*x*+1, −*y*, −*z*+1; (ii) *x*, −*y*+1/2, *z*+1/2; (iii) *x*+1, −*y*+1/2, *z*+1/2; (iv) *x*−1, *y*, *z*.
